# Angiogenic Potential of Human Bone Marrow‐Derived Mesenchymal Stem Cells in Chondrocyte Brick‐Enriched Constructs Promoted Stable Regeneration of Craniofacial Cartilage

**DOI:** 10.5966/sctm.2016-0050

**Published:** 2016-09-14

**Authors:** Zhiye Li, Ruikai Ba, Zhifa Wang, Jianhua Wei, Yimin Zhao, Wei Wu

**Affiliations:** ^1^ Department of Oral and Maxillofacial Surgery, State Key Laboratory of Military Stomatology, National Clinical Research Center for Oral Diseases, and Shaanxi Key Laboratory of Stomatology, School of Stomatology, Fourth Military Medical University, Xi'an, People's Republic of China; ^2^ Department of Prosthodontics, State Key Laboratory of Military Stomatology, National Clinical Research Center for Oral Diseases, and Shaanxi Key Laboratory of Stomatology, School of Stomatology, Fourth Military Medical University, Xi'an, People's Republic of China

**Keywords:** Adult human bone marrow, Angiogenesis, Chondrogenesis, Cell transplantation, Bone marrow stromal cells

## Abstract

Craniofacial deformities caused by congenital defects or trauma remain challenges for clinicians, whereas current surgical interventions present limited therapeutic outcomes. Injection of bone marrow‐derived mesenchymal stem cells (BMSCs) into the defect is highly desirable because such a procedure is microinvasive and grafts are more flexible to fill the lesions. However, preventing hypertrophic transition and morphological contraction remain significant challenges. We have developed an “all host derived” cell transplantation system composed of chondrocyte brick (CB)‐enriched platelet‐rich plasma (P) gel and BMSCs (B). Without exogenous biomaterials or growth factors, such grafts regenerate cartilage efficiently and present great clinical promise. In immunodeficient mice, we compared performance of BMSCs and BMSCs lacking angiogenic potential in CB‐B‐P constructs and followed the cartilage maturation process by histology, immunostaining, micro‐computed tomography, and protein analysis. We determined that angiogenesis occurred quickly inside rudimentary cartilage derived from CB‐B‐P constructs after implantation, which improved tissue survival, tissue growth, and production of chondrogenic signals from chondrocytes. In contrast, silencing angiogenic potential of BMSCs led to poor chondrogenesis accompanied by necrosis. Chondrocyte bricks merged rapidly with angiogenesis, which constituted an enclosed chondrogenic niche and effectively inhibited runt‐related transcription factor‐2‐dependent hypertrophic transition of BMSCs as well as endochondral ossification; progressive chondrogenic differentiation of BMSCs resulted in vascularization regression, thus favoring persistent chondrogenesis and effectively augmenting nasal cartilage. In conclusion, these findings provided a novel, efficient approach to regenerating cartilage tissues in vivo. Chondrocyte bricks mixed with P provide transient vascularization and a persistently chondrogenic microenvironment for BMSCs; this provides a mini‐invasive approach for craniofacial cartilage reconstruction. Stem Cells Translational Medicine *2017;6:601–612*

**Significance Statement**

Craniofacial deformations caused by congenital defects or trauma remain challenge for clinicians. Injection of bone marrow‐derived mesenchymal stem cells (BMSCs) into the defect is highly desirable for repair of craniofacial deformations because such a procedure is microinvasive and grafts are more flexible to fill the lesions with various shapes. This study assessed an all‐host‐derived tissue engineering construct. By harnessing a fragmented chondrocyte‐cartilaginous extracellular matrix (chondrocyte bricks [CBs]), platelet‐rich plasma (P) could be formulated and enforced. This CB‐P system provided an angiogenesis‐favorable niche for seeded BMSCs. This study showed the angiogenic potential of BMSC‐enabled CB‐B‐P constructs to recapitulate the early development event in rudimentary cartilage–transient vascularization. It also proved that transient vascularization is crucial for stable cartilage formation, including morphology and cartilaginous phenotype. This can be used as a novel approach to apply mesenchymal stem cells in craniofacial cartilage repair.

## Introduction

Craniofacial deformities caused by congenital defects or trauma remain challenges for clinicians. Owing to morphologically variant lesion, injection of bone marrow‐derived mesenchymal stem cells (BMSCs) into the defect is highly desirable for cartilage repair because such a procedure is microinvasive and grafts are more flexible to fill the lesions with arbitrary shapes. However, preventing the hypertrophic transition of MSCs and morphological contraction in vivo remain significant challenges for injectable grafts 1, 2. Recapitulating embryological development is becoming a more attractive approach to engineer organ or three‐dimensional (3D) tissue from stem cells 3. Chondrogenesis is the process by which cartilage forms from condensed mesenchyme tissue 4. For therapeutic cartilage regeneration, harvesting mesenchymal stem cells (MSCs) from bone marrow and reconstituting them into cell aggregate with pellet or transwell culture have been widely studied for cartilage regeneration 5. Benefiting from gap junction‐mediated intercellular contacts and interactions between cell‐extracellular matrix (ECM) 6, chondrogenic differentiation of BMSCs in an aggregating model was significantly enhanced by exogenous growth factors as compared with solid scaffold‐based cell transplants 7. Therefore, development of injectable cell macroaggregate may offer an approach that could crack the bottle neck of cartilage regeneration by using stem cells.

The key to promoting in vivo cartilage formation of 3D premature BMSC aggregates is intrinsic and appropriate signaling stimuli 8. The 3D chondrogenic niche is expected to provide morphologically supportive and persistent chondrogenic stimulus for stem cells 9. Hydrogels chemically modulated with signaling molecules are therefore developed to provide a 3D niche with persistently chondrogenic cues for seeding cells 10, 11. However, in vivo behaviors remain far from satisfactory owing to inflammatory reactions, interior necrosis, and hypertrophic ossification 12. Coculturing approaches to tissue engineering are still a promising way to reduce donor cartilage and thus hold great promise for clinical translation; constructs are often fabricated by direct sediment of stem/progenitor cells in scaffolds or into micromass pellets 13. Coculturing chondrocytes and BMSCs in a polymeric scaffold successfully repaired articular cartilage in animals, which showed antihypertrophic phenotypes and produced adequate amount of glycosaminoglycans 14, 15, 16. In addition to the paracrine effect of chondrocytes on the coimplanted BMSCs, antiangiogenic factors released from chondrocytes are thought to play an essential role in persistent chondrogenesis of BMSCs 17, 18. A seeding mixture of chondrocytes and BMSCs in an ear‐shaped polyglycolic acid scaffold generated elastic cartilage with precise auricular shape in SCID mice 19. However, long‐term in vitro incubation and prolonged presence of foreign materials in vivo significantly reduced safety and reliability when applied in clinic. Developing “all host derived” grafts may solve the preceding problems and are more clinically translational.

Harnessing self‐produced ECM of chondrocytes may endow grafts with intrinsic mechanical stiffness and bioactive signals 20, 21. To provide injectable, mechanical supportive BMSC aggregate for cartilage regeneration, we developed a chondrocyte brick (CB; fragmented chondrocyte/ECM macroaggregates)‐enriched platelet‐rich plasma (PRP) gel. A previous study showed that mixing chondrocytes with cell bricks/PRP stabilized clots efficiently 22. In this study, we found that undifferentiated BMSCs could be well mixed with chondrocyte bricks in a PRP gel and self‐assembled into a macroaggregate of a size that can be used clinically. Furthermore, such a completely cellularized graft not only was acquired throughout tissue survival but also presented persistent chondrogenesis with a determined outline. Further mechanistic investigation of these findings is essential to reveal interactions between BMSCs and chondrocyte bricks and to identify their respective roles in tissue survival and persistent chondrogenesis. We found angiogenic potential in undifferentiated BMSCs vascularized CB constructs shortly after transplantation, which prevented interior necrosis, promoted merging of chondrocyte bricks, and rapidly increased compressive resistance of grafts. Furthermore, merging CBs constituted an enclosed chondrogenic niche, effectively inhibited runt‐related transcription factor‐2 (RUNX‐2)‐dependent hypertrophic transition of BMSCs, and prevented endochondral ossification, thus favoring persistent chondrogenesis and augmenting nasal cartilage with precise morphology.

## Materials and Methods

All experimental protocols involving human cells and animal experiments were approved by the Ethics Committee of the Fourth Military Medical University. Human BMSCs (hBMSCs) were obtained from patients undergoing iliac bone grafting surgery, with informed consent. Bone marrow was flushed out of iliac cancellous bone and dispersed with 40 ml of Dulbecco's modified Eagle's medium (DMEM; HyClone, GE Healthcare Life Sciences, Logan City, UT, https://promo.gelifesciences.com/gl/hyclone/) and centrifuged at 1,500 rpm for 5 minutes, followed by resuspension in DMEM‐low glucose supplemented with 10% fetal bovine serum (FBS; HyClone, GE Healthcare Life Sciences), 50 μg/ml penicillin and 30 μg/ml streptomycin (Amresco, Cleveland, OH, https://www.amresco‐inc.com). Primary cells were seeded in a 75‐cm^2^ culture flask at 5.0 × 10^5^ nucleated cells per cm^2^ in DMEM (high glucose; HyClone, GE Healthcare Life Sciences) containing 10% FBS and incubated at 37°C with 5% CO_2_. The nonadherent cells were removed during the first medium change. The adherent cells were incubated until cell clones reached greater than 80% confluence; cells were then digested with 0.25% trypsin (HyClone, GE Healthcare Life Sciences) and subcultured at 1.0 × 10^4^ cells per cm^2^. Half the passage 3 cells were transfected with lentivirus vectors containing small hairpin RNA targeting human vascular endothelial growth factor (VEGF)—sense,5′‐GATCCC(G)CCATGAACTTTCTGCTGTCTTGATATCCGGACAGCAGAAAGTTCATGGTTTTTTCCAAC‐3′; antisense, 3′‐GG(C)GGTACTTGAAAGACGACAGAACTATAGGCCTGTCGTCTTTCAAGTACCAAAAAAGGTTGAGCT‐5′ (Genomeditech, Shanghai, China, http://www.genomeditech.com) at multiplicity of infection of 10 plaque‐forming units per cell with 5 μg/ml Polybrene (Sigma‐Aldrich, St. Louis, MO, http://www.sigmaaldrich.com), which was used as VEGF‐silencing BMSCs (BMSCs^VEGF−^) for further experiment. The other half of these cells were transfected with empty lentivirus vectors at the same concentration with the groups mentioned.

Human auricular chondrocytes (hACs) were isolated from the microtia specimens during ear reconstruction procedures, with informed consent. Briefly, isolated primary hACs were incubated in DMEM‐high glucose (HyClone, GE Healthcare Life Sciences) containing 10% FBS (HyClone, GE Healthcare Life Sciences) for proliferation. After two passages, two thirds of hACs were cultured at 6.5 × 10^5^ cells per cm^2^ in 6‐cm‐diameter culture dishes in DMEM‐high glucose supplemented with 20% FBS (HyClone, GE Healthcare Life Sciences), l‐glutamine (272 μg/ml; Amresco), ascorbate 2‐phosphate (50 μg/ml; Sigma‐Aldrich), and 50 μg/ml penicillin and 30 μg/ml streptomycin (Amresco) and incubated at 37°C with 5% CO_2_. After 12–15 days, when the solid white membrane of chondrocytes formed, the membranes were fragmented in a homemade cutting system to obtain chondrocyte bricks by following the procedure described previously 21. One third of passage 2 hACs were cultured in 25‐cm^2^ culture flasks at 1.5 × 10^5^ cells per cm^2^ in DMEM‐high glucose (HyClone, GE Healthcare Life Sciences) containing 10% FBS (HyClone, GE Healthcare Life Sciences) to enhance proliferation for 12–15 days.

### Transwell Chemotaxis Assay

Human umbilical vein endothelial cell (HUVEC) migration was performed in a transwell chemotaxis 24‐well chamber (Corning Glassworks, Corning, NY, http://www.corning.com). hBMSCs, chondrocytes, and CBs cultured for 2 weeks were used in this experiment. Eight groups were set according to cell seeding in the lower chamber (a) basal medium (as a negative control); (b) BMSC group: A total of 3 × 10^4^ hBMSCs transfected with empty lentivirus were added in the lower wells of transwell inserts; (c) chondrocytes (C) group: A total of 3 × 10^4^ chondrocytes were added in the lower wells of transwell inserts (d) CB group: chondrocyte bricks formed from cultured 3 × 10^4^ chondrocytes were added in the lower wells of transwell inserts; (f) BMSC^VEGF−^ group: A total of 3 × 10^4^ VEGF‐silencing BMSCs were added in the lower wells of transwell inserts; (g) C + BMSC group: A total of 3 × 10^4^ hBMSCs transfected with empty lentivirus and 3 × 10^4^ chondrocytes were added in the lower wells of transwell inserts; (h) CB + BMSC group: A total of 3 × 10^4^ hBMSCs transfected with empty lentivirus and chondrocyte bricks formed from 3 × 10^4^ chondrocytes were coseeded in the lower wells of transwell inserts; (i) CB + BMSC^VEGF−^ group: A total of 3 × 10^4^ VEGF‐silencing hBMSCs and chondrocyte bricks formed from 3 × 10^4^ chondrocytes were coseeded in the lower wells of transwell inserts.

Nine hundred microliters of medium containing the preceding groups of cells was added to each of the lower wells; in addition, HUVECs were suspended with 100 µl FBS‐free endothelial basal medium containing 1% FBS (2 × 10^5^ cells per ml) and then added to upper wells of transwells in triplicate. After 12 hours of incubation, nonmigrated HUVECs in the upper wells were removed from the filter by washing three times with phosphate‐buffered saline (PBS) and gentle scraping with cotton swabs. HUVECs that migrated to the underside of the membrane were fixed with methanol for 15 minutes and stained with 0.1% crystal violet (Sigma‐Aldrich). A stereo microscope (SZX16‐3141, Olympus, Tokyo, Japan, http://www.olympus‐global.com) was used to take the general photos, and a light microscope was used to count the number of cells on the underside of the insert for each condition. Five fields were counted for each well (original magnification, ×200) to estimate the mean number of cells for each replicate. Migrated cells were quantified by Image‐Pro Plus 6.0 (Media Cybernetics, Bethesda, MD, http://www.mediacy.com).

### Condition Medium Collection and Enzyme‐Linked Immunosorbent Assay

To evaluate secretory VEGF from all groups of cells, we set the following groups: (a) BMSC: 1 × 10^5^ hBMSCs transfected with empty lentivirus; (b) C + BMSC: A total of 1 × 10^5^ hBMSCs transfected with empty lentivirus and 3 × 10^4^ chondrocytes were cultured; (c) CB + BMSC: A total of 1 × 10^5^ hBMSCs transfected with empty lentivirus and chondrocyte bricks formed from 3 × 10^4^ chondrocytes were coseeded; (d) CB + BMSC^VEGF−^: A total of 1 × 10^5^ VEGF‐silencing hBMSCs and chondrocyte bricks formed from 3 × 10^4^ chondrocytes were coseeded. These cells were seeded in 6‐well plates and cultured for 24 hours; the medium was replaced with 2 ml DMEM containing 10% FBS for an additional 48 hours. Collected medium was centrifuged (2500 rpm for 3 minutes) to remove cell debris and then used for experiments. Enzyme‐linked immunosorbent assay (ELISA) kits (Sigma‐Aldrich) were used to quantify VEGF concentration in medium from each group according to the manufacturer's instructions. Additionally, seeded cells from each group were harvested for protein extraction and analysis by Western blot.

### Animal Experiments

Thirty‐nine nude mice were used for in vivo experiments. The chondrocyte‐BMSC‐PRP (C‐B‐P) group, chondrocyte brick‐BMSC‐PRP (CB‐B‐P) group, and chondrocyte brick‐VEGF silencing BMSC‐PRP (CB‐B^VEGF−^‐P) group were set for abdominally subcutaneous injection (33 of these mice). The animals were sacrificed for sample harvest at 2 weeks, 4 weeks, and 12 weeks after graft implantation (*n* = 3 at 2 weeks and *n* = 4 for the other groups). A sample of 500 μl PRP was used per animal. BMSCs, BMSCs^VEGF−^, chondrocytes, and chondrocyte bricks were collected and rinsed with PBS once. For the C‐B‐P group, BMSCs (2.25 × 10^7^ cells) and chondrocytes (7.5 × 10^6^ cells) were centrifuged into a mixing pellet and resuspended with PRP (500 μl) sufficiently. For the CB‐B‐P and CB‐B^VEGF−^‐P groups, BMSCs (2.25 × 10^7^ cells)/BMSCs^VEGF−^ (2.25 × 10^7^ cells), and chondrocyte bricks (from 7.5 × 10^6^ primary cells) were mixed by using the same process.

The injection process was performed according to the following procedure: PRP‐cellular components as prepared per preceding method were aspirated into a 2‐ml syringe. The syringe was then aspirated with 50 μl of a thrombogenic agent (100 U/ml in 100 mg/ml calcium chloride; Biomedical Technologies, Villalba, Madrid, http://www.biomedical‐technologies.com/) and mixed. After nude mice were anesthetized through inhalation of diethyl ether, the clotting constructs were injected subcutaneously into the abdominal site of nude mice via a 16‐guage needle. The construct turned into a gel‐like spheroid immediately after injection. To characterize the chondrocyte bricks enriched with PRP gel, some pieces of complex were fixed in 2.5% glutaraldehyde and then processed for examination with scanning electronic microscopy (JSM6330F; Jeol, Akishima, Tokyo, Japan, http://www.jeol.co.jp). Additionally, 6 other nude mice were used for an animal functional model constructed by subcutaneously injecting CB‐B‐P/CB‐B^VEGF−^‐P (300 μl PRP, 6.75 × 10^6^ cells BMSCs or BMSCs^VEGF−^, chondrocyte bricks from 4.5 × 10^6^ primary cells) to the nasal dorsum of animals.

### Gross Morphology

We measured the wet weight, thickness, and volume of the samples, which were dissected from the surrounding tissues 12 weeks after injection, as described elsewhere 22. Specimens and the constructs for functional experiment were also evaluated for contour deformation.

### Micro‐Computed Tomography

Micro‐computed tomography (CT) was performed on the head of each functional model animal as well as constructs subcutaneously injected into the abdomen after 12 weeks of in vivo incubation. We reconstructed the image data by using NRecon software, version 1.5.1.4 (Bruker microCT, Kontich, Belgium, http://bruker‐microct.com) to visualize the 3D representation of the constructs to compare the areas of ossification in constructs of three groups and to analyze differences among groups. Micro‐CT software (Inveon Research Workplace, Siemens, Germany, https://www.healthcare.siemens.com) was used to calculate the percentage of ossifying volume to total construct volume (BV/TV) of each sample.

### Glycosaminoglycan and Collagen Quantification

Collagen content was quantified as described previously with a Sircol Collagen Assay (S1000; Biocolor, Carrickfergus, U.K., http://www.biocolor.co.uk). Briefly, parts of the 4‐ or 12‐weeks samples were cut, minced into 1‐mm^3^ pieces, and then digested with pepsin (1 mg/ml in 0.5 M acetic acid; MP, Biomedicals, Santa Ana, CA, http://www.mpbio.com/), the obtained supernatant was used for testing samples according to kit instructions. Collagen standard curve was calculated, from which total collagen per construct wet weight was obtained.

Sulfated glycosaminoglycans (sGAGs) quantification was processed by using a Blyscan sGAG Assay Kit (B1000; Biocolor) as described elsewhere 21. Briefly, sGAG was extracted from tiny pieces of specimens at 4 or 12 weeks after injection after being digested with papain extraction (Sigma‐Aldrich) reagent at 65°C for 18 hours. Measurement of sGAG concentration in the supernatant was then performed according to the kit instructions, followed by calculation of sGAG per wet weight construct from the sGAG standard curve.

### Histological and Immunohistochemical Assay

Samples were fixed in 4% paraformaldehyde, embedded in paraffin, and then cut into 6‐μm sections. Hematoxylin and eosin, safranin‐O, Masson trichrome, and Von Kossa stains were performed as standard procedures. Furthermore, expressions of type I and type X collagen and VEGF in constructs were investigated via two‐step indirect immunohistochemical staining. Briefly, immunohistochemical staining was processed with primary anticollagen type I antibody (mouse anti‐human, 1:50; Abcam, Cambridge, U.K., http://www.abcam.com), anticollagen type II antibody (mouse anti‐human, 1:200; OriGene Technologies Inc., Rockville, MD, http://www.origene.com), anti‐collagen type X antibody (mouse anti‐human, 1:100; Abcam), and anti‐VEGF antibody (mouse anti‐human, 1:100; Abcam), followed by horseradish peroxidase‐conjugated anti‐mouse antibody (1:200 in PBS; Santa Cruz Biotechnology, Dallas, TX, http://www.scbt.com) and color development with diaminobenzidine tetrahydrochloride (Santa Cruz Biotechnology).

### Immunofluorescence Assay

To analyze the vasculature of constructs, parts of samples were cryosectioned into 8‐μm sections without fixing. The sections were permeabilized with 0.3% Triton X‐100 at room temperature (RT) for 20 minutes, followed by being blocked with 5% goat serum for 20 minutes at RT. Then the samples were incubated with anti‐CD31 antibody (mouse anti‐human, 1:100; Abcam) at 4°C overnight. After incubation with Cy3‐AffiniPure conjugated secondary antibody (goat anti‐mouse, 1:100; Jackson ImmunoResearch Laboratories, West Grove, PA, https://www.jacksonimmuno.com/) at RT for 2 hours, the sections were incubated with 4′,6‐diamidino‐2‐phenylindole (Qcbio, Shanghai, China, http://www.qcbio.company.weiku.com) for 5 minutes. Finally, the sections were observed and photographed under a fluorescent microscope (Olympus IX71).

### RNA Isolation and Real‐Time Polymerase Chain Reaction

One‐step phenol chloroformisoamyl alcohol extraction of total RNA was processed for human microtia auricular cartilage as native control and different groups at two time points by RNAiso Plus (Takara Bio Inc., Shiga, Japan, http://www.takara‐bio.com) as described by the manufacturer's protocol. Real‐time reverse‐transcription polymerase chain reaction (RT‐PCR) analysis of five genes (*SOX‐9*, *RUNX‐2*, *VEGF*, *Collagen X,* and *GAPDH*) was performed and replicated five times by using One Step SYBR PrimeScript RT‐PCR Kit (Takara Bio Inc.), the primer sequences used in this study are listed in supplemental online Table 1. The 2^‐ΔΔCt^ method was used to compare differences of gene expression among the three groups.

### Western Blot

Harvested fresh samples were grinded in liquid nitrogen and lysed mammalian protein extraction reagent (Thermo Fisher Scientific Life Sciences, Waltham, MA, http://www.thermofisher.com) supplemented with complete protease inhibitor. BCA Protein Assay Kit (Thermo Fisher Scientific Life Sciences) was used to determine total protein contents, which were adjusted to 10 μg/μl. Ten‐microliter samples were loaded onto 10%–14% SDS polyacrylamide gels and transferred to a polyvinylidene fluoride membrane (EMD Millipore, Billerica, MA, http://www.emdmillipore.com/). The membranes were blocked by using 5% skim milk in 0.05% Tris‐buffered saline/Tween 20 for 1 hour. The membrane was then incubated at 4°C overnight with the following primary antibodies: anti‐SOX‐9 (mouse anti‐human, 1:500; Abcam), anti‐RUNX‐2 (mouse anti‐human, 1:200; Abcam), anti‐VEGF (mouse anti‐human, 1:200; Abcam), anti‐collagen type X (mouse anti‐human, 1:500; Abcam), anti‐β‐actin (1:1,000; Cell Signaling, Danvers, MA, https://www.cellsignal.com/), respectively. After further washes, chemiluminescence of the blot was processed with the enhanced chemiluminescence Western blotting substrate (ThermoFisher Scientific Life Sciences).

### Statistical Analysis

All quantitative results are expressed as mean ± SD. Statistical analyses were performed by using SPSS software, version 17.0 (IBM, Chicago, IL, http://www.ibm.com). Analysis of variance was used for multiple group comparisons. The Tukey honestly significant difference test was used for pairwise comparisons. Two‐tailed *p* values <.05 were considered to indicate statistically significant differences.

## Results

### hBMSCs Aggregated Spontaneously in Chondrocyte Brick‐Enriched PRP Gel

**Figure 1:**
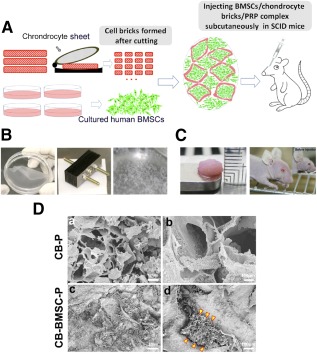
Characterization of CB‐based injectable cell aggregate. **(A):** Schematic depiction of the strategy for CB‐based cell transplantation and in vivo implantation. **(B):** A chondrocyte sheet was cultured, harvested, and embedded for fragmenting in a net cutting system (by multiple blades). **(C):** The CBs or chondrocytes and cultured BMSCs were mixed together or BMSCs alone were suspended in PRP, so that the injectable constructs were formed and injected subcutaneously into nude mice. **(Da, Db):** Scanning electron microscopy images show that CBs mixed with PRP gel formed small cavities and connected with each other. **(Dc):** BMSCs filled in cavities among chondrocytes bricks. **(Dd):** BMSCs aggregated among CBs and reconstituted into macroaggregate along with CBs (arrowheads). (**[Da, Dc]:** low magnification, scale bars = 1 mm; **[Db, Dd]:** high magnification, scale bars = 100 μm). Abbreviations: BMSC, bone marrow‐derived mesenchymal stem cells; CB, chondrocyte brick; P and PRP, platelet‐rich plasma.

Figure 1A depicts the construction and implantation of the CB‐B‐P constructs. Figure 1B shows that the harvested chondrocyte sheets were cut by using a homemade cutting device, which was composed of multiple blades so that 1‐mm^2^ fragments were obtained. Cell‐ECM fragments with such a size can pass smoothly through a16‐guage needle, thus fulfilling a criterion for injection. After transfection with empty lentivirus vectors, cultured hBMSCs were mixed with chondrocyte bricks and then clotted with PRP, resulting in morphologically stable constructs and significantly inhibited shrinkage, which efficiently supported constructs subcutaneously after injection into the nasal dorsum. Macroscopic examination of constructs after in vivo implantation confirmed that injected constructs maintained the appropriate volume and outline subcutaneously (Fig. 1C). Scanning electron microscopy images of injectable constructs showed that chondrocyte bricks served as a framework inside the PRP gel and formed multiple, enclosed cavities prepared for BMSC filling (Fig. 1Da, 1Db). Spindle cells filled in spaces among bricks and presented as a cell cluster, with ideal cell‐cell contact (Fig. 1Dc, 1Dd).

### Proangiogenic Potential of hBMSCs Was Maintained When Cocultured With Chondrocytes or Chondrocyte Bricks

**Figure 2:**
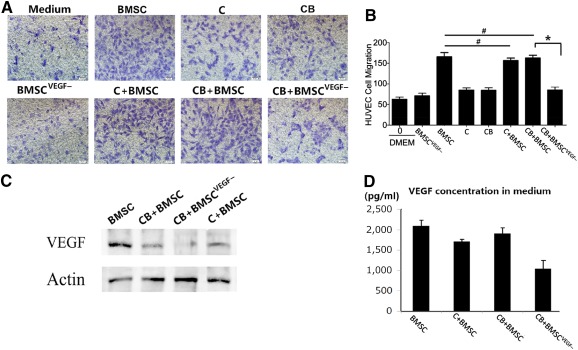
Proangiogenic role of BMSCs in coculturing system in vitro. **(A):** Representative images showed cell migration of HUVECs after coculturing with conditioned medium derived from BMSCs, C, CBs, BMSC^VEGF−^, C + BMSC, CB + BMSC, and CB + BMSC^VEGF−^, respectively. Scale bars = 50 μm. **(B):** Bar graph shows quantitative analysis of cell migration of HUVECs in each group; *n* = 4, ∗, *p <* .05; #, *p >* .05. **(C):** Protein expression of VEGF and actin by Western blot in BMSC, CB + BMSC, CB + BMSC^VEGF−.^, and C + BMSC. **(D):** Detection of VEGF concentration by using enzyme‐linked immunosorbent assay in conditioned medium of BMSC, C + BMSC, CB + BMSC, CB + BMSC^VEGF−^; *n* = 4 for each group. Abbreviations: BMSC, bone marrow‐derived mesenchymal stem cells; BMSC^VEGF−^, vascular endothelial growth factor‐silencing bone marrow‐derived mesenchymal stem cells; C, chondrocyte; CB, chondrocyte brick; DMEM, Dulbecco's modified Eagle's medium; HUVEC, human umbilical vein endothelial cell; VEGF, vascular endothelial growth factor.

To further evaluate angiogenic potential of BMSCs when they were cocultured with chondrocytes in vitro, we compared effects of coculturing BMSCs/chondrocytes, BMSCs/chondrocyte bricks, and BMSCs^VEGF−^/chondrocyte bricks on endothelial (HUVEC) migration by using a transwell system. As shown in Figure 2A and 2B, HUVEC migration was active in all BMSC (transfected with empty lentivirus vectors)‐involved groups, no significant difference was detected among these groups, and mixing chondrocytes with BMSCs did not inhibit HUVEC migration (*p* > .05). In contrast, VEGF silencing in BMSCs significantly reduced HUVEC migration (*p* < .05). By using quantification tests, including ELISA of proteins extracted from culture medium and Western blot for proteins lysated from cultured cells, VEGF concentration presented in culturing medium only slightly decreased while remaining at the same level for all BMSC‐involved groups (Fig. 2C, 2D) (*p* > .05). VEGF silencing via lentivirus transduction downregulated VEGF production and reduced the proangiogenic potential of cells. These results confirmed the proangiogenic properties of BMSCs when these cells were cocultured with endothelial cells. Together, these findings indicated that coseeding of BMSCs significantly enhanced angiogenic potential of constructs and was not influenced by antiangiogenic property of chondrocytes.

### Angiogenic Potential of hBMSCs Induced Early Vascularization in Constructs

**Figure 3:**
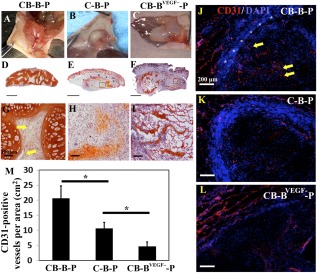
Sprouting of new blood vessels from the host to the engineered tissue grafts 2 weeks after implantation. **(A–C):** Macroscopic appearances of constructs after 2 weeks in vivo. **(D–F):** Safranin‐O staining of grafts 2 weeks after implantation (scale bars = 2 mm). **(G):** Orange staining depicts the CBs; spindle cells among CBs are biomaterial seeded with mesenchymal stem cells. Yellow arrows, host vessel sprouting to the constructs. **(G–I):** High magnification of images showing details; yellow arrows, new capillaries (original magnification, ×20; scale bars = 100 μm). **(J–L):** Representative images of CD31‐stained blood vessels (red) in grafts at 2 weeks after implantation. **(J):** CB‐B‐P grafts. **(K):** C‐B‐P grafts. **(L):** CB‐B^VEGF−^‐P grafts. For J–L, the nuclei are stained blue (scale bar = 200 μm). **(M):** Vascularization quantification of CB‐B‐P versus C‐B‐P or CB‐B^VEGF−^‐P constructs. The density of CD31‐positive vessels was measured at 2 weeks after implantation. All values are normalized to the graft area (cm^2^). ∗, *p <* .05. For all determinations, the sample size was *n* = 3, and all values are represented as mean ± SEM. Abbreviations: B, bone marrow‐derived mesenchymal stem cells; B^VEGF−^, vascular endothelial growth factor‐silencing bone marrow‐derived mesenchymal stem cells; C, chondrocyte; CB, chondrocyte brick; DAPI, 4′,6‐diamidino‐2‐phenylindole; P, platelet‐rich plasma.

After transplantation in vivo, constructs were harvested at the second week for angiogenesis examination. Macroscopically, within 2 weeks of implantation, all grafts appeared viable and had become vascularized to a varied extent. Capillaries were observed in the capsule surrounding the grafts, suggesting that the constructs had integrated with the host tissue (Fig. 3A–3C). CB‐B‐P grafts were most highly vascularized (Fig. 3J), as indicated by the mean capillary density of CD31‐positive vessels per cm^2^ and significantly higher than that observed in the C‐B‐P and CB‐B^VEGF−^‐P grafts (Fig. 3K, 3L). Early vessel infiltration nourished CB‐B‐P constructs, which vitalized most of the tissue in the construct (Fig. 3D). Chondrocyte bricks in CB‐B‐P constructs appeared significantly enlarged and merged with each other, and histological images showed opening circular neovessels formed in the BMSC aggregate, which indicated that blood perfusion had been established (Fig. 3G). In contrast, the CB‐B^VEGF−^‐P group presented poor growth of chondrocyte bricks and necrotic cavities in constructs (Fig. 3F). The C‐B‐P constructs presented as a contracted, condensed cellular mass (Fig. 3E); despite the vascular infiltration in the C‐B‐P constructs, the average vessel number of several fields was significantly lower than that in the CB‐B‐P constructs (Fig. 3I).

Quantitatively, the average number of blood vessels in the CB‐B‐P and C‐B‐P constructs (20.0 ± 1.9 vessels per cm^2^ and 12.7 ± 3.1 vessels per cm^2^) was significantly higher than that in the CB‐B^VEGF−^‐P group (5.0 ± 1.1 vessels per cm^2^; *p* < .05). More interestingly, despite the vascular infiltration in the C‐B‐P constructs, the average vessel number of several fields was significantly lower than that in the CB‐B‐P constructs (*p* < .05) (Fig. 3M).

### Vascularization Promoted Cartilaginous Remodeling of Constructs

After 12 weeks, samples in the CB‐B‐P group turned into white, elastic cartilage‐like tissue with a smooth surface (supplemental online Fig. 1A), whereas constructs from the C‐B‐P and CB‐B^VEGF−^‐P groups had a rough appearance and irregular outline (supplemental online Fig. 1C, 1E), and the C‐B‐P constructs presented hardening, ossified parts. Wet weight of the CB‐B‐P construct (351.7 ± 27.1 mg) was significantly larger than those of the C‐B‐P (115.2 ± 7.2 mg; *p <* .05) and CB‐B^VEGF−^‐P group (130.2 ± 12.9 mg; *p <* .05) (supplemental online Fig. 1H). Accordingly, volume measurement also indicated significant differences among the groups (*n* = 4; *F* = 517.5; *p <* .05) (supplemental online Fig. 1G), the CB‐B‐P constructs (361.7 ± 28.6 μl) was significantly higher than those of the C‐B‐P group (193.3 ± 12.1 μl; *p <* .05) group and CB‐B^VEGF−^‐P group (226.3 ± 20.2 μl; *p <* .05). Moreover, thickness of samples varied significantly (*n* = 4; *F* = 29.65; *p <* .05) (supplemental online Fig. 1I). The mean thickness of the CB‐B‐P constructs (4.0 ± 0.3 mm) was significantly higher than those of the C‐B‐P (3.0 ± 0.2 mm; *p <* .05) group and CB‐B^VEGF−^‐P group (3.2 ± 0.2 mm; *p <* .05). Micro‐CT‐based BV/TV quantification showed that ossifying components in all samples was significantly different (*n* = 4; *F* = 516.2; *p < .*05) (supplemental online Fig. 1J), which demonstrated little ossification in CB‐B‐P constructs (5.2% ± 0.4%; *p <* .05), significant ossification in C‐B‐P constructs (46.3% ± 3.6%; *p <* .05) and partial ossification in CB‐B^VEGF−^‐P constructs (12.1% ± 1.1%; *p <* .05).

**Figure 4:**
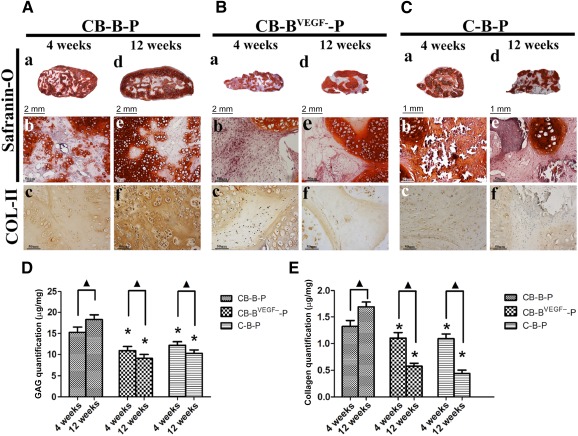
Evaluation of cartilage formation for CB‐B‐P, C‐B‐P, and CB‐B^VEGF‐^‐P constructs. **(A–C):** Histological analysis of chondrogenesis of grafts in vivo after 4 weeks (left lane of **[A–C]**) and 12 weeks (right lane of **[A–C]**). Merged images (Safranin‐O staining) showed that the CB‐B‐P and C‐B‐P groups acquired tissue survival throughout, whereas CB‐B^VEGF‐^‐P group was characterized by central necrosis and poor tissue merging. The CB‐B‐P group presented good chondrogenic differentiation in the biomaterial seeded with mesenchymal stem cell regions, as was confirmed by Safranin‐O and COL‐II immunostaining **(Aa–Af)**. In contrast, progressive osteogenesis and calcification occurred in the C‐B‐P group **(Ca–Cf)**. Scale bars = 2 mm **(Aa Ad, Ba, Bd)**, 1 mm **(Ca, Cd)**, and 50 μm **(Ab, Ac, Ae, Af, Bb, Bc, Be, Bf, Cb, Cc, Ce, Cf)**. **(D, E):** Quantitative evaluation of cartilaginous extracellular matrix, including glycosaminoglycans and collagen. ∗, *p* < .05 compared with the same time points of CB‐B‐P constructs; ▲, *p* < .05 for comparision of the same group between 4 and 12 weeks (*n* = 4 in each group). Abbreviations: B, bone marrow‐derived mesenchymal stem cells; B^VEGF−^, vascular endothelial growth factor‐silencing bone marrow‐derived mesenchymal stem cells; C, chondrocyte; CB, chondrocyte brick; P, platelet‐rich plasma.

We further analyzed the cartilaginous remodeling at the histological level. The cartilaginous character of regenerated tissue was examined by safranin‐O staining and type II collagen immunostaining. For CB‐B‐P constructs, after the fourth week, chondrocyte bricks merged with each other and connected into a net‐like framework, and BMSCs filled in small cavities (Fig. 4Aa, 4Ad). Higher magnification presented light GAG staining of BMSC regions and deep‐stained mature chondrocyte bricks, which indicated chondrogenic differentiation of BMSCs in constructs (Fig. 4Ab, FAe). Until the 12th week, the deep GAG staining presented in mature cartilaginous regions significantly expanded, and net‐like chondrocyte bricks further thickened and merged; this resulted in formation of a thick cartilaginous shell peripherally in the constructs (Fig. 4Ad, 4Ae). Immunostaining confirmed that COL‐II, a crucial cartilaginous component, was increasing in BMSC regions of CB‐B‐P constructs, which indicated stable chondrogenesis of BMSCs and chondrocyte brick constructs (Fig. 4Ac, 4Af).

GAG quantification showed increasing cartilaginous components and indicated active chondrogenesis in CB‐B‐P constructs (Fig. 4D). In contrast, reduction of the proangiogenic potential of BMSCs led to significantly poor chondrogenesis of constructs. After 4 weeks’ in vivo incubation, CB‐B^VEGF−^‐P constructs presented a compacted structure and necrotic cavities in interior of constructs (Fig. 4Ba); safranin‐O‐stained chondrocyte bricks were distributed separately in constructs and were mixed with collapsed and contracted BMSC aggregate (Fig. 4Bb). The 12‐week constructs presented less cartilaginous tissue formation in constructs, which was characterized by poor merging, less expanding chondrocyte bricks, and significantly less safranin‐O staining in BMSC regions. Additionally, necrotic cavities remained in constructs and were accompanied by collapsed structure (Fig. 4Bd, 4Be). In addition, Col‐II immunostaining confirmed weak production of such cartilaginous components (Fig. 4Bc, 4Bf). In C‐B‐P samples, two distinct tissue regions were presented as early as the fourth week: chondrocyte brick‐derived cartilaginous tissue that was strongly positive for GAG staining and BMSC‐derived ossifying ECM (Fig. 4Ca–4Cc). Furthermore, 12 weeks of in vivo incubation resulted in cancellous bone and mature cartilaginous matrices from chondrocyte bricks (Fig. 4Cd–4Cf).

Collagen and sGAG were extracted and examined quantitatively (Fig. 4D, 4E). Both collagen and glycosaminoglycan contents within samples revealed significant differences among groups (4 weeks, *n* = 4: collagen, *F* = 210.5, *p* < .05; sGAG, *F* = 117.2, *p* < .0001; 12 weeks, *n* = 4: collagen, *F* = 36.54, *p* < .05; sGAG, *F* = 115.5, *p* < .05). The CB‐B‐P constructs presented the highest cartilaginous ECM production compared with average levels in the other two groups through weeks 4–12. From 4 to 12 weeks, the CB‐B‐P constructs presented an increasing amount of collagen and GAG content (4 weeks: collagen, 1.30 ± 0.11 μg/mg; sGAG, 15.21 ± 1.13 μg/mg, 12 weeks: collagen, 1.65 ± 0.17 μg/mg; sGAG, 17.89 ± 1.67 μg/mg); although these amounts were lower than those in native cartilage (collagen, 2.11 ± 0.20 μg/mg; sGAG, 19.62 ± 1.31 μg/mg), they were significantly higher than those in the C‐B‐P and CB‐B^VEGF−^‐P group. Interestingly, C‐B‐P constructs presented decreasing glycosaminoglycan production from 4 to 12 weeks, and silencing VEGF in BMSCs resulted in significantly less GAG production compared with the other two groups; at the same time, collagen production was slightly enhanced. This result stressed the determining role of chondrocyte bricks in preventing ossification. Moreover, the augmented morphological stability of BMSC grafts was paralleled by enhanced VEGF expression and was abolished by VEGF‐silence treatment on BMSCs. This finding suggests an important role for proangiogenic potential of BMSCs in stable tissue remodeling and cartilage regeneration.

### Vascularization of BMSC Aggregate in CB‐P Constructs Was Transient

**Figure 5:**
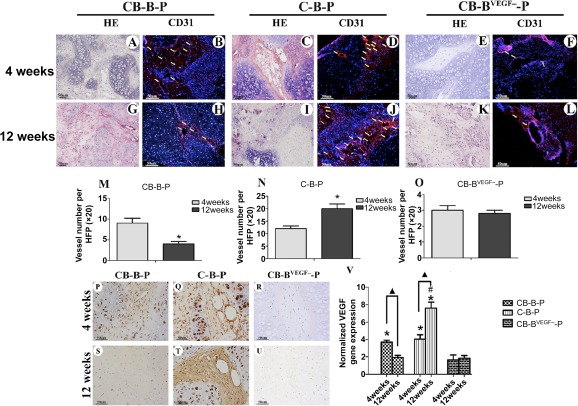
Evaluation of angiogenesis at weeks 4 and 12. Representative images of CB‐B‐P, C‐B‐P and C‐B^VEGF‐^‐P constructs revealed infiltration of capillaries at week 4 **(A, C, E)** and 12 **(G, I, K)**, which was predominantly distributed in bone marrow‐derived mesenchymal stem cells (BMSC) regions, as confirmed by CD31 immunofluorescent staining (4 weeks: **[B, D, F]**; 12 weeks: **[H, J, K]**); scale bars = 50 μm. **(M–O):** Comparison of the number of blood vessels between weeks 4 and 12; the value represents the average number of all fields examined (*n* = 6). **(P–U):** Immunostaining of VEGF reveals decreasing expression of VEGF in BMSC regions through 4 and 12 weeks in the CB‐B‐P group, whereas increasing expression in the C‐B‐P group, in addition, C‐B^VEGF‐^‐P constructs showed weak VEGF expression; scale bars = 50 μm. **(V):** Real‐time polymerase chain reaction quantitatively revealed the expression profile of VEGF in all groups. ∗, *p* < .05 compared with CB‐B^VEGF−^‐P at the same time point, #, *p* < .05 compared with CB‐B‐P at week 12; triangles, *p* < .05 for comparision of the same group between 4 and 12 weeks (*n* = 4 in each group). Abbreviations: B, bone marrow‐derived mesenchymal stem cells; B^VEGF−^, vascular endothelial growth factor‐silencing bone marrow‐derived mesenchymal stem cells; C, chondrocyte; CB, chondrocyte brick; HE, hematoxylin and eosin; HPF, high‐power field; P, platelet‐rich plasma; VEGF, vascular endothelial growth factor.

Vascular infiltration determined nutritional perfusion of transplanted cells, especially for those in the interior of grafts. Both C‐B‐P and CB‐B‐P constructs presented full cellularization and tissue survival throughout the constructs. To determine the reason for this, we monitored spatio‐temporal angiogenesis in these constructs. Histologically, in BMSC regions dispersed among avascular chondrocyte bricks, round, monolayer capillary structures were widely distributed throughout the constructs after the 4th week (Fig. 5A), as was confirmed with CD31 immunostaining (Fig. 5B). Furthermore, we recorded the vascular amount in the 4th and 12th week (Fig. 5B, 5H) by counting CD31‐positive vessels and measuring their area according to a previously published procedure 18. Interestingly, vessel number in the CB‐B‐P group significantly decreased from the 4th to the 12th week (8.2 ± 1.7 vessels per high‐power field vs. 4.1 ± 1.2 vessels per high‐power field; *p* < .05) (Fig. 5M). In contrast, vessel numbers in the C‐B‐P group kept increasing (Fig. 5C, 5D, 5I, 5J, 5N); this increase was also accompanied by marrow reconstruction. In contrast, significantly less vascularization occurred in the CB‐B^VEGF−^‐P group (Fig. 5E, 5F, 5K, 5L, 5O), and a few vessels could be found peripherally in constructs. According to these results, angiogenesis analysis in this part proved that vascularization of BMSC aggregate in CB‐P constructs is transient, which is different from BMSC‐chondrocyte mixing constructs.

We further evaluated the variation of the angiogenic potential of BMSCs in constructs. VEGF, a key component to induce angiogenesis, was evaluated by using immunostaining and real‐time RT‐PCR. In accordance with angiogenesis in BMSC regions, the CB‐B‐P group and C‐B‐P group both expressed moderate VEGF after week 4 (Fig. 5P, 5Q). As a control, VEGF expression in the BMSCs^VEGF−^ groups persistently remained at a low level (Fig. 5R, 5U). Interestingly, VEGF immunostaining in CB‐B‐P constructs significantly decreased from weeks 4 to 12 (Fig. 5P, 5S) and was completely different from the persistent angiogenesis seen during endochondral ossification of the C‐B‐P samples (Fig. 5Q, 5T). Real‐time RT‐PCR confirmed the expression of VEGF mRNA in the BMSC regions of the constructs (Fig. 5V). Although the expression of VEGF was significantly higher than that of native cartilage, it decreased in CB‐B‐P at week 12 as compared with week 4 (*p* < .05) (Fig. 5V); it even decreased to the same level as that of native cartilage (*p* > .05). In contrast, at week 12, BMSCs in the C‐B‐P group displayed higher expression of VEGF than did the CB‐B‐P samples (*p* < .05) (Fig. 5V), and VEGF‐silencing BMSCs persistently presented lower VEGF expression at week 4 and slight enhancement at week 12 (Fig. 5R, 5U, 5V). These results indicated that the angiogenic potential of BMSCs varied in different coimplantation systems and that chondrocyte bricks could significantly regulate angiogenesis of BMSCs after the fourth week in vivo.

### Transient Vascularization Did Not Lead to Ossification of Constructs

**Figure 6:**
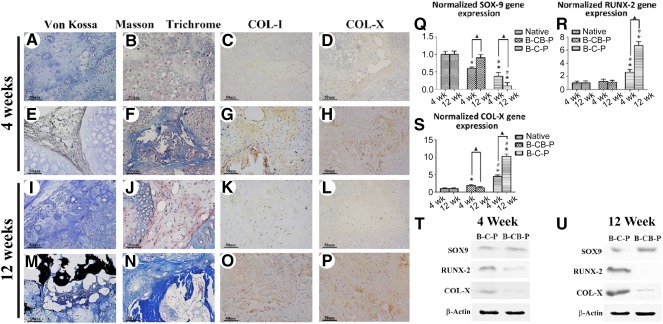
Osteogenesis of grafts in vivo at 4 weeks **(A–H)** and 12 weeks **(I–P)**. The CB‐B‐P group did not present ossification of BMSCs through 12 weeks **(A–D; I–L)**, as was confirmed by Von Kossa, Masson trichrome, COL‐I, and COL‐X immunostaining. In contrast, BMSCs in C‐B‐P presented early calcification, and progressive ossification occurred through 12 weeks **(E–H, M–P)**; scale bars = 50 μm. Quantitative analysis of osteogenic markers including SOX‐9 **(Q)**, RUNX‐2 **(R)**, and COL‐X **(S)** by using real‐time polymerase chain reaction and Western blotting **(T, U)**. ∗, *p* < .05 compared with native cartilage at the same time point; #, *p* < .05 compared with CB‐B‐P at the same time point; ▲, *p* < .05 for comparision of the same group between 4 and 12 weeks (*n* = 4 in each group). Abbreviations: B, bone marrow‐derived mesenchymal stem cells; C, chondrocyte; CB, chondrocyte brick; P, platelet‐rich plasma; RUN‐X, runt‐related transcription factor‐2.

Hypertrophic differentiation of BMSCs and ossification are always important issue that hinder application of BMSCs in ectopic chondrogenesis, which frequently accompanies vascularization. We therefore analyzed the effect of vascularization on the final translation of constructs. Constructs from the CB‐B‐P and C‐B‐P groups were stained with Von Kossa, Masson trichrome, and immunostaining for COL‐I and COL‐X. Just as Figure 6E and 6M shows, Von Kossa staining revealed calcium, shown as black, widely deposited crystals, and indicated that hypertrophic transition rapidly occurred in the C‐B‐P group as early as 4 weeks after surgery (Fig. 6E); significantly more calcium could be found in the 12‐week group (Fig. 6M), which confirmed that vascularized bone tissue was formed in B‐C‐P constructs. Masson trichrome staining of samples demonstrated production of mature collagen in the C‐B‐P and CB‐B‐P groups (Fig. 6B, 6F, 6J, 6N); however, further immunostaining revealed that Col‐I and Col‐X, characterized components of ossifying tissue, were positively stained in C‐B‐P constructs (Fig. 6G, 6H, 6O, 6P), whereas staining in CB‐B‐P constructs is faint (Fig. 6C, 6D, 6K, 6L). Furthermore, the expressions of hypertrophic markers, RUNX‐2 and COL‐X, in the C‐B‐P group were significantly higher than those in the CB‐B‐P group and native cartilage through 12 weeks (*p* < .05) (Fig. 6R, 6S). Activation of RUNX‐2 and downstream molecules, including COL‐I and COL‐X, indicated poor antihypertrophic ability in C‐B‐P constructs. In contrast, in the CB‐B‐P constructs, inhibition of RUNX‐2 was detected after week 4 and even extended to week 12 (Fig. 6R).

Moreover, the expression of SOX‐9, known as a chondrogenesis marker, increased significantly in the CB‐B‐P group, which was even the same as the expression in native cartilage after 12 weeks (Fig. 6Q). In contrast, the expression of SOX‐9 in the C‐B‐P group decreased and was significantly lower than that in the CB‐B‐P group and native cartilage after 12 weeks. Western blot analysis showed that the expression of hypertrophic proteins, such as RUNX‐2 and COL‐X, in the C‐B‐P group increased with time and was higher than CB‐B‐P group through 12 weeks (Fig. 6T, 6U). The preceding results indicated that upregulation of SOX‐9 and downregulation of RUNX‐2 of BMSCs were regulated by chondrocyte bricks, which affected the angiogenesis and hypotrophy of BMSCs. Because of the increasing regulation of vascularization of BMSCs in the microenvironment by VEGF, which was affected by the balance of SOX‐9 and RUNX‐2, the CB‐B‐P construct maintained persistent ectopic chondrogenesis and inhibited excess angiogenesis. This, in turn, led to hypertrophy and ossification.

### Vascularized Constructs Enhanced Mechanical Resistance and Augmented Nasal Dorsum Efficiently

**Figure 7:**
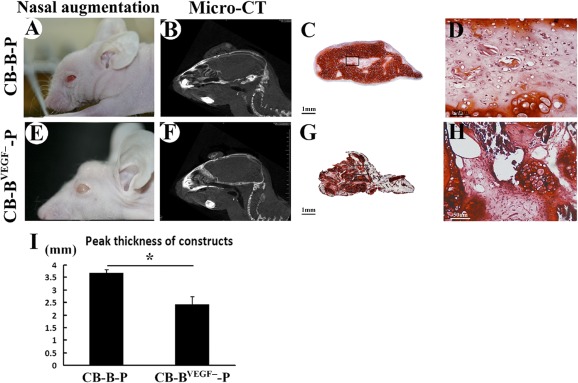
Nasal augmentation by injecting chondrocyte bricks enriched constructs. **(A, E):** Macroscopic appearance of nasal dorsum at week 12 after injection of CB‐B‐P and CB‐B^VEGF−^‐P. **(B, F):** Examination of injected constructs by micro‐computed tomography. **(C, D):** Safranin‐O staining of the cross‐sections presented cartilage‐like tissue without ossification and higher magnification for above staining. **(G, H):** Safranin‐O staining for samples from CB‐B^VEGF−^‐P constructs. Scale bars = 1 mm **(C, G)**, 50 μm **(D, H)**. **(I):** Quantitative evaluation of peak thickness between CB‐B‐P and CB‐B^VEGF−^‐P constructs. ∗, *p* < .05 (*n* = 3 in each group). Abbreviations: B, bone marrow‐derived mesenchymal stem cells; B^VEGF−^, vascular endothelial growth factor‐silencing bone marrow‐derived mesenchymal stem cells; C, chondrocyte; CB, chondrocyte brick; CT, computed tomography; P, platelet‐rich plasma.

In contrast to the abdominal region, nasal dorsal skin underwent consistent abrasion and compression and therefore could be used to test the compression‐resistant property of the grafts. We investigated the role of early vascularization in the enhancing ability of morphological maintenance. After 300 μl of gel‐like CB‐B‐P complex was injected subcutaneously into the nasal dorsum, the skin was elevated and gelling substances filled the subcutaneous cavity according to the customized nasal morphology. Slight volume reduction could be observed during the further in vivo remodeling process, which resulted in significant augmentation of the nasal dorsum (Fig. 7A). In contrast, loss of proangiogenic potential of BMSCs resulted in compressed morphology, and augmentation was less efficient (Fig. 7E). Micro‐CT quantitatively showed volume and thickness of samples (Fig. 7B, 7F). In accordance with gross appearance, peak thickness of CB‐B^VEGF−^‐P constructs (2.42 ± 0.3 mm) significantly reduced in comparison with CB‐B‐P constructs (3.66 ± 0.14 mm; *p* < .05) (Fig. 7I).

We further analyzed the histological presentation of these samples (Fig. 7C, 7D, 7G, 7H). Safranin‐O staining showed that most of the constructs turned into glycosaminoglycan‐enriched ECM, with significant merging of fragmented chondrocytes. Sparsely distributed immature cartilaginous tissue filled central regions. CB‐B^VEGF−^‐P constructs, however, showed collapsed microstructure and chondrocyte bricks compacted together; merging was poor, which explained poor augmentation in vivo. These results demonstrated that the angiogenic potential of BMSCs in CB‐B‐P constructs significantly influenced compression‐resistant ability during in vivo remodeling, thereby supporting regeneration of nasal cartilaginous tissue in craniofacial regions.

## Discussion

For reconstructive surgeries for craniofacial deformities, precise morphology is the most important requirement of grafts 23. Our study showed that artificial inhibition of the angiogenic potential in BMSCs makes CB‐B‐P constructs transform into a collapsed, contracted mass; lack of persistent chondrogenic signals in C‐B‐P constructs also led to contracted and ossified tissue that thus failed to augment the nasal dorsum. Our findings demonstrate that three features of the reported graft are vital for craniofacial cartilage reconstruction: early vascularization, adequate mechanical support, and persistent chondrogenic signals. We suspect that early vascularization is the most crucial because it connects the grafts with the host body quickly and thus allows tissue survival. Neoangiogenesis progressively offers more oxygen and nutrition for cell proliferation, differentiation, and matrix production. Adequate mechanical support may further promote vascularization and host remodeling, which is likely to warrant stable space for vascular cell infiltration and avoid cell condensation or tissue collapse.

Platelet‐rich plasma, a natural medium for tissue healing, has presented superior angiogenic ability and avoids host exposure to foreign material 24. Long‐lasting materials cause tissue stiffening from fibrous encapsulation and can activate inflammatory cells to induce scar formation 25. Chondrocyte bricks are a primary component of our grafts; these are different from chondrocytes. Preformed ECM in bricks offers intrinsic mechanical stiffness of constructs and thus compensates for the weak stiffness of PRP. Finally, chondrogenic signals released from chondrocytes directed chondrogenic differentiation of BMSCs, which may occur via a paracrine effect. With adequate nutritional provision, cell viability is warranted for producing chondrogenic factors.

The decisive angiogenic factors are growth factors released from cellular components in constructs, as has been shown by central necrosis in the chondrocyte‐alone group but full survival in the CB‐B‐P group. The angiogenic potential of BMSCs has been identified and applied for revascularization of ischemic heart and limbs in patients; secretory VEGF produced from transplanted BMSCs played a critical role in inducing angiogenesis 26, 27. However, whether or not the angiogenic potential of BMSCs benefits chondrogenesis remains debatable owing to the avascular nature of cartilage 28, 29. Use of chondrocytes/chondrocyte bricks/PRP or silencing‐VEGF expression of BMSCs both caused central malnutrition in constructs, which presented as a necrotic cavity, limited GAG production, and poor morphological maintenance. On the basis of this observation, VEGF secreted from BMSCs was believed to contribute to vascularization of the constructs and rapidly nourished the constructs. Because of its avascular nature, many antiangiogenic proteins have been purified from extracts of adult cartilage 30, which inhibits angiogenesis from surrounding vessels. Interestingly, in vitro examination showed that chondrocytes or chondrocyte bricks in our study did not decrease the proangiogenic ability of cocultured BMSCs. Our results confirm that expression of VEGF in undifferentiated BMSCs is critical for guiding endothelial cell migration, which initiated early and fast revascularization of whole construct. The above observation also indicated chondrocyte brick‐enriched PRP provided a favorable microenvironment for angiogenesis of undifferentiated BMSCs.

The unique feature of our cartilage graft is that it is "all host derived” and is based on a novel chondrocyte brick‐enriched PRP system. Compared with a solid scaffold, PRP gel is quickly degradable and weak in mechanical strength. The significant advantage of a PRP gel is that it enabled rapid tissue remodeling, which simulated the wound healing process through clot‐mediated vascular invasion. However, it is the weak property that resulted in contraction of the cell‐PRP gel construct, which inevitably led to host exposure of BMSCs. In vivo results showed that endochondral ossification started from BMSCs adjacent to surrounding host tissues in the C‐B‐P constructs. An opening microenvironment caused by fast degradation of peripheral PRP failed to retain paracrine factors released from chondrocytes, which resulted in progressive ossification of BMSCs. In contrast, with incorporation of chondrocyte bricks, the mechanical strength of constructs was enhanced by ECM produced in vitro from chondrocytes. Moreover, we found that progressive growth and merger of chondrocyte bricks in constructs guaranteed mechanical and biological stability of the graft. An enclosed cartilaginous shell formed as early as week 4 and thus provided an enclosed chondrogenic microenvironment. Moreover, it was the early vascularization that enabled further merging of chondrocyte bricks, as well as connection between BMSCs and chondrocyte bricks; thus, the merged cells persistently provided intrinsic support for constructs with the degradation of PRP and helped maintain the morphology 31.

The most interesting phenomenon occurring in chondrocyte brick‐based constructs is that the vascularization was transient, as confirmed by the observations that constructs grew through 4–12 weeks. This is different from the progressive angiogenesis and endochondral ossification in C‐B‐P constructs. This finding suggests the need for further investigation of different molecular events occurring in BMSCs from CB‐B‐P and C‐B‐P constructs. During endochondral ossification, chondrocyte hypertrophy is accompanied by an upregulation of collagen X, matrix metalloproteinases, and VEGF, all of which are targets of RUNX‐2 32.

Using real‐time RT‐PCR and Western blotting, we showed that mixing BMSCs with chondrocyte bricks significantly downregulated RUNX‐2 expression through weeks 4–12, whereas SOX‐9, a critical chondrogenic marker 33, was upregulated slowly. In contrast, the BMSC‐alone construct was completely transformed into bone tissue at this time. This finding demonstrated that chondrocyte bricks released factors influencing RUNX‐2/SOX‐9 balance, which inhibited osteogenic transition of MSCs and activated the chondrogenic pathway at the same time. It has been proved that BMSCs present decreased angiogenic potential once they are chondrogenically differentiated, and vascular regression is an essential event in persistent chondrogenesis 34, 35. Accordingly, in vivo results confirmed that vessel density decreased at week 12, and VEGF expression was significantly reduced after chondrogenic differentiation of BMSCs in a chondrocyte brick‐based microenvironment.

Of note, no necrosis was seen, despite the depression of capillaries. We believe that once chondrogenesis began, BMSCs were more resistant to hypoxia, thus guaranteeing whole tissue survival in constructs. This finding confirmed that the angiogenic potential of BMSCs is determined by differentiating stages of cells and that chondrocyte brick‐PRP actually provide a vascularization‐tunable microenvironment in which BMSCs could communicate with the host body freely and regulate capillary growth instantly.

## Conclusion

The findings reported here provide a novel, efficient and injectable approach to regenerate cartilage tissues in vivo. Chondrocyte bricks mixed with PRP provide a vascularization‐tunable and persistently chondrogenic microenvironment for BMSCs. VEGF expression in BMSCs is vital for early vascularization of construct and tissue growth, which contributes to the maintenance of morphology. Chondrocyte bricks did not influence the early angiogenic potential of undifferentiated BMSCs, but promoted persistent chondrogenesis without hypertrophic transition through regulating SOX‐9/RUNX‐2 balance. Such a microinvasive approach could be applied to nasal augmentation and other craniofacial cartilage reconstruction.

## Author Contributions

Z.L.: collection and/or assembly of data, data analysis and interpretation, manuscript revision; R.B.: collection and/or assembly of data, data analysis and interpretation, manuscript writing; Z.W.: data analysis and interpretation, manuscript writing; J.W.: collection and/or assembly of data, data analysis and interpretation; Y.Z.: conception and design, final approval of manuscript; W.W.: conception and design, manuscript writing, final approval of manuscript, financial support.

## Disclosure of Potential Conflicts of Interest

The authors indicated no potential conflicts of interest.

## Supporting information

Supporting Information
