# Supplementary material for: Angiogenic Potential of Human Bone Marrow‐Derived Mesenchymal Stem Cells in Chondrocyte Brick‐Enriched Constructs Promoted Stable Regeneration of Craniofacial Cartilage
Source: Stem Cells Transl Med. 2016 Sep 14;6(2):601–12. doi: 10.5966/sctm.2016-0050 (PMC5442805; doi:10.5966/sctm.2016-0050)
Supplement: Supplementary file 1 — Supporting Information [file SCT3-6-601-s001.pdf]

Supplemental Information – Wei Wu et al.

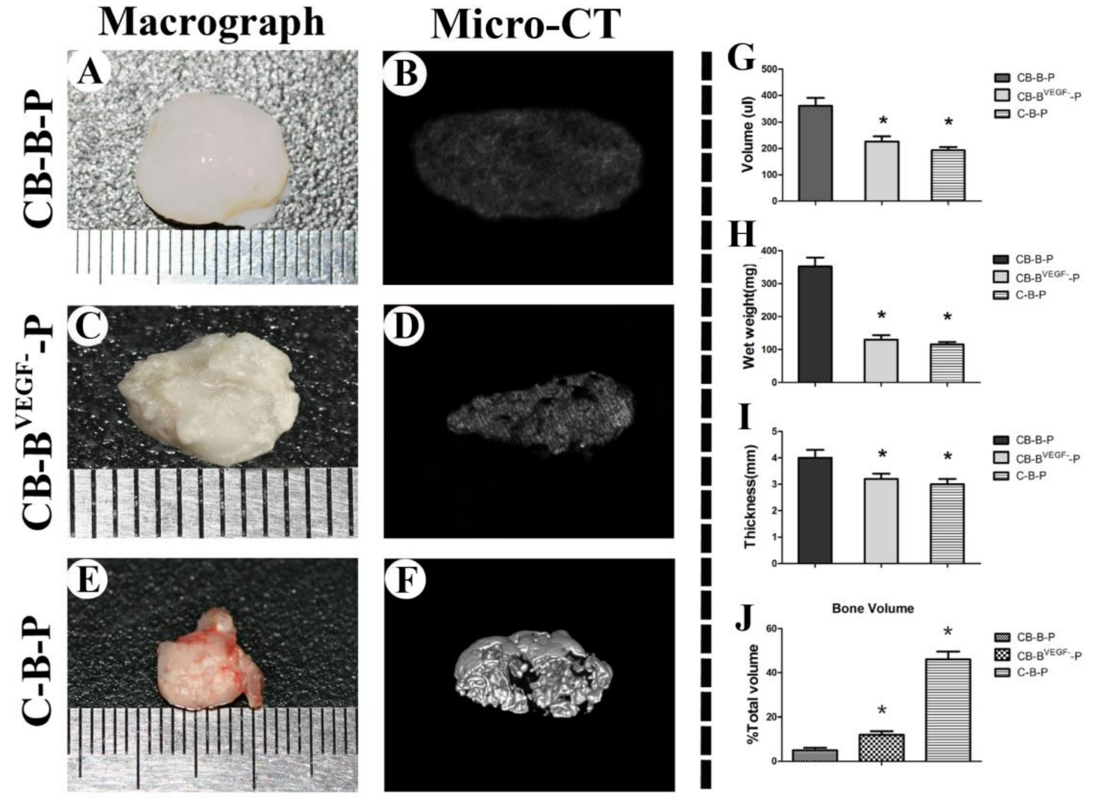

Figure S1. Morphological evaluation of CB-B-P, CB-B^{VEGF-}-P and C-B-P constructs after 12 weeks' *in vivo* incubation. **(A)**, **(C)** and **(E)** presented representative appearances of specimens from each group. **(B)**, **(D)** and **(F)** showed Micro-CT scanning for these samples. Quantitative measurement of samples presented different volume **(G)**, weight **(H)** and thickness **(I)**, and ossified tissue percentage based on CT analysis **(J)**, n=4, * denotes $p<0.05$.

Table S1 Gene primer sequence for Real time RT-PCR

Genes	Primers
SOX-9	Forward 5′ -AATCTCCTGGACCCCTTCAT-3′
	Reverse 5′-GTCCTCCTCGCTCTCCTTCT-3′
RUNX-2	Forward 5 ′-CGGAATGCCTCTGCTGTTAT-3′
	Reverse 5′-TTCCCGAGGTCCATCTACTG-3′
Collagen-X	Forward 5 ′-GGGATGCCTCTTGTCAGTGC-3′
	Reverse 5′-ATCTTGGGTCATAGTGCTGCTG-3′
VEGF	Forward 5 ′- ATCGAGACCTTGGTGGAC-3′
	Reverse 5′- CCTGGTGAGGTTTGATCC-3′
GAPDH	Forward 5 ′-TGGTATCGTGGAAGGACTCATGAC-3′
	Reverse 5′-ATGCCAGTGACGTTCCCGTTCAGC-3′
